# Frugal day-ahead forecasting of multiple local electricity loads by aggregating adaptive models

**DOI:** 10.1038/s41598-023-42488-1

**Published:** 2023-09-22

**Authors:** Guillaume Lambert, Bachir Hamrouche, Joseph de Vilmarest

**Affiliations:** 1 Électricité de France, R &D, 91477 Palaiseau, France; 2 Viking Conseil, 75007 Paris, France

**Keywords:** Energy infrastructure, Energy grids and networks, Power stations, Electrical and electronic engineering, Mathematics and computing, Statistics

## Abstract

This paper focuses on day-ahead electricity load forecasting for substations of the distribution network in France; therefore, the corresponding problem lies between the instability of a single consumption and the stability of a countrywide total demand. Moreover, this problem requires to forecast the loads of over one thousand substations; consequently, it belongs to the field of multiple time series forecasting. To that end, the paper applies an adaptive methodology that provided excellent results at a national scale; the idea is to combine generalized additive models with state-space representations. However, extending this methodology to the prediction of over a thousand time series raises a computational issue. It is solved by developing a frugal variant that reduces the number of estimated parameters: forecasting models are estimated only for a few time series and transfer learning is achieved by relying on aggregation of experts. This approach yields a reduction of computational needs and their associated emissions. Several variants are built, corresponding to different levels of parameter transfer, to find the best trade-off between accuracy and frugality. The selected method achieves competitive results compared to individual models. Finally, the paper highlights the interpretability of the models, which is important for operational applications.

## Introduction

Electricity consumption forecasting is essential for numerous activities: managing the electricity network, investment and production planning, trading on the electricity markets, and reducing power wastage. To suit these activities, forecasts are performed at different horizons, from short-term (hours, days) to long-term (years), and on different scales, from individual to national. New variability in the electricity load has recently emerged due to several factors: new decentralized production units (like solar panels), new actors with the opening of the French electricity market, new uses (like electric mobility), and the COVID-19 pandemic. They bring essential changes in the electricity consumption, and forecasting models must be updated to take them into account.

Numerous forecasting approaches have been proposed to forecast electricity consumption and the Global Energy Forecasting Competitions (GEFCom) provides an overview^1–3^. These methods include classical time series approaches such as auto-regressive integrated moving-average (ARIMA)^4,5^ and exponential smoothing^6^ that have been used to forecast at the very-short term (hours-ahead) or other statistical and machine learning approaches such as Gradient Boosting^7,8^ and Neural Networks^9,10^ that provide better forecasts using exogeneous variables, especially for larger horizons. Indeed, judging that the electricity demand is a human activity, it can be predicted using explanatory variables, the most important ones being weather and calendar data. In particular, Generalized Additive Models (GAMs)^11^ have been widely applied to forecast the electricity consumption^12–15^, yielding good results.

More recently, a state-space approach has been investigated to adapt GAMs over time^16^; this online model allows GAMs to adapt to new variabilities, such as the COVID-19 pandemic, to improve forecasts^17,18^. Finally, the variety of models motivates predicting the demand with a combination of forecasters, yielding a better final prediction than any individual model; that is the aggregation of experts^19^, which has also shown good results on load forecasting^20^. Aggregation of experts is an online method which combines predictions given by experts in a weighed sum where the weights evolve over time.

This paper investigates day-ahead electricity load forecasting on the distribution network in France. The electricity consumption is measured on about 2,200 substations located at the frontier between the high voltage grid and the distribution network. More precisely, data of 1344 of them is accessible, enabling local level forecasting. Forecasting multiple time series is an important task in many industries like retail, car sharing or electricity management. Several competitions have tackled this problem such as the ASHRAE competition on forecasting multiple buildings’ energy consumption^21^ or the Makridakis competitions^22,23^ leading to interesting findings. Among which, one of the most important being the importance of cross-learning from multiple time-series to improve accuracy. As underlined in the findings of these competitions, forecasting multiple time series is usually tackled using two kinds of approaches^24^: local methods that estimate one model independently for each time series and global methods that fit a unique model jointly for all of them. Local methods have been used the most for these problems in the past but global approaches have been shown to perform just as well especially when increasing model complexity^25^. In particular an extensive comparison between the two approaches has been tested on forecasting buildings’ consumption^26^. Computationally the two approaches face different costs and issues^26^: local approaches require time to train all the models but are easily parallelized. However, the cost of producing forecasts from trained models requires saving, maintaining and updating a large number of models which can be a hindrance. On the other hand, global approaches are harder to parallelize but are easier to maintain since only one model will be required at the end. Global approaches have the added advantage of being useful for forecasting new time series for which no data is available. This paper builds-up on a local approach^13^ while aiming to alleviate the computational and maintenance constraints associated with it. To that end, a trade-off must be found between accuracy and the amount of parameters and computational complexity.

Computational efficiency is a longstanding research issue in optimization^27^; the objective is to get a desired estimation in the shortest amount of time, or to get the best estimation in a fixed amount of time. An interesting subfield is online optimization^19,28^, where the objective is to use streaming data to update a model in a recursive and efficient manner. While computational time and costs remain important incentives for efficient machine learning methods, other motivations have gained attention in the recent years: energy consumption for environmental goals^29^, sparsity to improve interpretability^30^, and use of data to respect privacy or to avoid costly data collection or transmission^31,32^. This trade-off between performances and efficiency has previously been referred to as frugal machine learning^32^. In the case study of this paper, the original forecasting method learns an individual model for each time series, and therefore its computational time and energy consumption grow linearly with the number of time series considered. Reducing the time of the learning process has operational advantages; also, it paves the way to an application to even more local consumption (up to the extreme of individual households), leading to more numerous time series, in which case the learning process would become infeasible.

**Figure 1:**
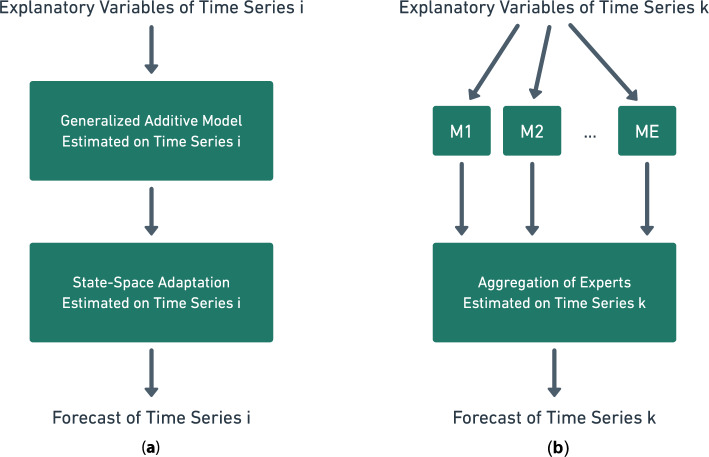
Transfer learning through aggregation of experts in two steps: (**a**) individual models are trained for *E* time series for a small *E* compared to the number of time series, then (**b**) to forecast any time series *k*, models $M_1$, $M_2$, ..., $M_E$ are applied and all the resulting forecasts are combined using aggregation of experts.

To reduce the computational burden this paper adopts a transfer learning point of view represented with diagrams in Fig. 1. Individual models are trained on a few time series, relying on GAMs and their adaptive variant based on state-space models. Then, these models learned on a small fraction of the time series are transferred to all of them, instead of training an individual model for each time series, using aggregation of experts as a transfer tool. Precisely, the base models are applied on each time series even if they were not trained on them, and then aggregating these weak forecasters yields a procedure able to scale to a large number of time series. Several aggregations are built involving different kinds of models, where transfer learning occurs at multiple levels.

The paper discusses the computational cost for each aggregation type and focuses on their frugal nature. Indeed, optimization of individual models to a large number of time series is very costly while aggregation of experts is not. It is shown that the presented methods compete with individual GAMs, even during the first French lockdown due to COVID-19, while achieving low computational cost. Transfer learning through aggregation of experts has also been studied in a hierarchical setting, using regional load data as sources and national load data as target^33^.

Our contribution may be summarized as follows: building on local GAM models, we propose to use adaptive state-space models and transfer learning based on aggregation of experts to alleviate the constraints associated with local approaches (mainly in terms of maintenance and computing costs), leading to a frugal model that performs better than the individual models, with the added benefit of being usable on new time series with minimal historical depth. To that end, we first propose different ways to transfer a model learned on a time series to the others. Then, we introduce a way to use several models learned on several time series and combine them to forecast each time series. Finally, we apply the approach on the electricity load of French substations of the distribution network.

Following this introduction, the “Methodology” section establishes the methods used in this paper. The “Data and model presentation” section introduces the data and the models applied. The results of the forecasting tasks are analysed in the “Experiments” section. Finally, the “Conclusion” section summarises the paper and proposes future work.

## Methodology

This section first characterises the forecasting problem in the transfer learning framework. Then, the principles of the aggregation of experts are detailed along with its use for transfer learning. Finally, the different models considered in the paper, and the resulting aggregation methods, are introduced.

### Transfer learning context

The transfer learning approach taken in this paper can be expressed using the definitions and vocabulary used for transfer learning methods^34^. First, it is essential to provide the definition of domain and task. A domain ${\mathscr {D}} = \{ {\mathscr {X}}, P(X) \}$ is composed of a feature space ${\mathscr {X}}$ and a marginal distribution *P*(*X*) of an instance set $X = \{ x \mid x_i \in {\mathscr {X}}, i = 1, \dots , D \}$. A task ${\mathscr {T}} = \{ {\mathscr {Y}}, f \}$ is composed of a label space ${\mathscr {Y}}$ and a decision function *f*. Given $m_S \in {\mathbb {N}}^+$ source domains and tasks and a target domain and task, transfer learning is the use of the knowledge within the source domains and tasks to improve the performance of the target decision function.

Each substation represents one target whose decision function varies from the other substations’ decision functions. Thus, there are $m_T = 1344$ transfer learning tasks, each corresponding to one forecasting task. This results in a multi-task transfer learning situation. On the other hand, several sources are used to do the transfer, $m_S > 1$, resulting in a multi-source transfer learning method.

The investigated forecasting problem may be characterised as homogeneous and inductive in the transfer learning terminology. Indeed, a homogeneous transfer learning scenario occurs when the source and target feature spaces are equal, as well as the source and target label spaces. The explanatory variables (calendar and meteorological variables and past electricity load) are equally available for all substations and range thus in the same feature space. The mutual label space is ${\mathbb {R}}_+$ corresponding to the electricity measurement range. Concerning inductive transfer learning, it happens when labeled data are available in the target domain to induce the target decision functions, which is the present case.

### Transfer learning using aggregation of experts

A natural idea to apply transfer learning in this context could be to determine a subsample of $m_S$ data sets, build one individual model per data set, and select the best among the $m_S$ models for each of the $m_T$ substations to forecast. The information on the best model being unavailable; one way to estimate it is through the aggregation of experts.

In the aggregation of experts context^19^, the aim is to predict a bounded sequence of observations $y_1,\dots ,y_n \in [0,B]$ (*B* is unknown) using *E* forecasting models called experts. For each time step $t \in \{ 1,\dots ,n \}$, they provide *E* forecasts $(\hat{y}_t^e)_{e=1}^E$ of the observation $y_t$. The aggregation $\hat{y}_t = \sum _{e=1}^E \hat{p}_{e,t} \hat{y}_t^e$ is then computed where the weights $(\hat{p}_{e,t})_{e=1}^E$ are updated online according to past performances of each expert. Forecast error is measured with a convex loss function $\ell _t(y_t, \cdot )$. The goal is to minimise the so-called regret $R_T = \frac{1}{T} \sum _{t=1}^T \ell _t(y_t, \hat{y}_t) - \frac{1}{T} \sum _{t=1}^T \ell _t(y_t, \hat{y}_t^\star )$ where $\hat{y}_t^\star$ is given by an oracle model which can use unavailable information to build a forecast difficult to beat. The regret is the difference between the error suffered by the aggregation and the error of the oracle. The latter can be the best-fixed convex combination of all the experts or the best-fixed expert (constant over time). The used algorithm for the present work is the ML-Poly algorithm^35^, successfully applied for electricity load forecasting^36^ and implemented in the R package opera^37^. This algorithm tracks the best expert or the best convex combination of experts by giving more weight to an expert that will generate a low regret. This makes this algorithm particularly interesting as no parameter tuning is needed.

Transfer learning through aggregation of experts can be considered as a parameter-based transfer learning method. Indeed, the aim is to produce multiple source learner models, which are introduced in the next section, and combining them to create specific target learners.

### Adaptive experts

This paragraph details the individual models that will be aggregated. They are GAMs adapted by Kalman filtering as proposed in^17^. This section considers the forecasting of one time series.

#### Generalized additive models

Generalized additive models (GAM)^11^ assumes that the response variable $y_t$ is expressed as the following sum:

1 $$\begin{aligned} y_t = \beta _0 + \sum _{d = 1}^D f_d(x_{t,d}) + \varepsilon _t, \end{aligned}$$

where $\beta _0$ is an intercept, $\varepsilon _t$ is the model error at time *t*, $(x_{t,d})_{d=1}^D$ are the *D* explanatory variables available at time *t* and $(f_d)_{d=1}^D$ are linear or nonlinear smooth functions called GAM effects. An effect $f_d$ is expressed as a projection

2 $$\begin{aligned} f_d(x) = \sum _{k=1}^{m_d} \beta _{d,k} B_{d,k}(x), \end{aligned}$$

where $(B_{d,k})_{k=1}^{m_d}$ is a spline basis of dimension $m_d$ and $(\beta _{d,k})_{k=1}^{m_d}$ are the corresponding coefficients estimated by a ridge regression, where the following criterion is minimized:

3 $$\begin{aligned} \sum _{t=1}^T \left( y_t - \sum _{d=1}^{D} f_d(x_{t,d}) \right) ^2 + \sum _{d=1}^D \lambda _d \int \Vert f_d '' (x) \Vert ^2 dx. \end{aligned}$$

The penalty term controls the second derivatives $f_d ''$ to force the effects to be smooth. $C_1$ denotes the computational cost of GAM estimation.

#### Adaptation by Kalman filter

To adapt a GAM, a multiplicative correction of the following GAM effects vector $f(x_t) = (1, \overline{f}_1(x_{t,1}), \dots , \overline{f}_d(x_{t,d}))^\top$ is applied, where $\overline{f}_j$ is a normalized version of $f_j$ obtained by subtracting the mean on the train set and dividing by the standard deviation.

The adaptation is obtained assuming that a state-space property is satisfied. Precisely, a vector $\theta _t$ called state is estimated under the assumption that

4 $$\begin{aligned}&y_t = \theta _t^\top f(x_t) + \varepsilon _t\,, \end{aligned}$$

5 $$\begin{aligned}&\theta _{t+1} = \theta _t + \eta _t\,, \end{aligned}$$

where $(\varepsilon _t)$ and $(\eta _t)$ are Gaussian white noises of respective variance / covariance $\sigma ^2$ and *Q*.

Starting from a Gaussian prior and assuming the variances $\sigma ^2$ and *Q* are known, the Kalman filter^38^ achieves the estimation of the state $\theta _t$. This is a Bayesian method where at each step, the state posterior distribution is obtained as a Gaussian distribution $\theta _t\mid (x_s,y_s)_{s<t}\sim {\mathscr {N}}(\hat{\theta }_t,P_t)$.

The Kalman filter depends on the initial parameters $\hat{\theta }_1,P_1$ (the prior) and the variances $\sigma ^2$ and *Q*. These hyper-parameters are chosen by maximizing the likelihood on the training set, using an iterative greedy procedure described in^16^, chapter 5. This choice of hyper-parameters is referred as the *dynamic* setting. Its computational cost is noted $C_2$ and can be large; it is detailed in the experimental study. Thanks to transfer learning, the costly estimation of these hyper-parameters is avoided for each time series.

Another interesting setting, called *static*, is when $Q = 0$, $\sigma ^2=1$, $P_1 = I$ and $\hat{\theta }_1 = 0$. In that case, the state equation becomes $\theta _{t+1}=\theta _t$ and the estimate $\hat{\theta }_t$ is equivalent to the ridge forecaster:

6 $$\begin{aligned} \hat{\theta }_t = \mathop {\textrm{arg min}}\limits _{\theta \in {\mathbb {R}}^d} \left( \sum _{s=1}^{t-1} (y_s - \theta ^\top f(x_s))^2 + \Vert \theta \Vert ^2 \right) . \end{aligned}$$

#### Models considered

As previously mentioned, the estimation of the GAM and of the Kalman variances is computationally costly when applied to 1344 time series. On the other hand, the computational time due to Kalman updates can be neglected. The proposed method reduces the number of GAMs and Kalman variances that are estimated.

Hybrid experts ${\mathscr {M}}_{i,j,k}$ are thus defined, where the different parts of the forecaster are trained on different time series. GAM is trained on the data set *i*, Kalman variances optimized (in the dynamic setting) on the data set *j*, and resulting adapted model applied on the data set *k*, where *i*, *j*, and *k* range in $\{1,\dots ,m_T\}$. In particular, ${\mathscr {M}}_{i,\emptyset ,k}$ denotes a non-adapted GAM and ${\mathscr {M}}_{i,0,k}$ an adapted GAM in the static setting where no variances are optimized.

There are three basic models without transfer learning where the models are optimized on the same substation’s data to forecast: ${\mathscr {M}}_{k,k,k}$, ${\mathscr {M}}_{k,0,k}$, and ${\mathscr {M}}_{k,\emptyset ,k}$. It is the scenario of traditional machine learning. Three models involving transfer learning are considered. The most simple transfer is GAM transfer: a source data set is used to train the GAM, and the resulting GAM is applied to a different target data set. It corresponds to the model ${\mathscr {M}}_{i,\emptyset ,k},\ i \ne k$. An immediate improvement of the previous model is the adaptation of the transferred GAM with a Kalman filter optimized on the same data set used to train the GAM. It results in models ${\mathscr {M}}_{i,i,k}, \ i \ne k$. The adaptation step helps the GAM transfer and improves the basic GAM. Finally, the Kalman filter transfer case: the data set to forecast is used to train a GAM adapted by a Kalman filter optimized on another data set. These are models ${\mathscr {M}}_{k,j,k},\ j \ne k$.

### Aggregation models

As previously said, the estimation of Kalman variances in the dynamic setting is computationally costly (e.g. training of ${\mathscr {M}}_{k,k,k}$ model), and the goal is to avoid individual training for all time series. Moreover, although the computational cost of GAM effects estimation is smaller, it is desirable to train only a few of them to reduce the cost and the number of model parameters. To do so, three aggregation methods are built based on the three previous models where transfer learning occurs, provided in the “Models considered” section. AGG GAM TL is obtained from the aggregation of $n_1~{\mathscr {M}}_{i,\emptyset ,k}$ models, AGG GAM-Kalman TL from $n_2~ {\mathscr {M}}_{i,i,k}$ models, and AGG Kalman TL from $n_3~ {\mathscr {M}}_{k,j,k}$ models. The data sets used to train GAMs and Kalman filters in each aggregation method are randomly chosen among the 1344 data sets.

Table 1 details the three aggregation methods, and the two individual adapted GAMs. It specifies if a transfer is involved, the type of the model used, and the computational costs corresponding to GAM and Kalman variances estimation. Judging that GAM, Kalman filter, and aggregation applications are computationally cheap, they are overlooked. For each aggregation method, parameter $n_i$ is the unique hyper-parameter of the method and corresponds to its number of sources $m_S$. It must be as small as possible compared to $m_T = 1344$ to force the frugality of the method.

**Table 1:** Description of the individual adapted models and aggregation methods considered in the case study. $C_1$ and $C_2$ correspond to the computational costs of GAM and Kalman variances estimation, respectively. $m_T$ is the number of targets in the transfer learning context, and $n_i$ is the number of experts in the three aggregation methods. To give an order of magnitude, in our application, parameters $n_i$ are inferior to 10 while $m_T = 1344$.

Characteristics of adapted GAM	GAM + Kalman Static	GAM + Kalman Dynamic	AGG GAM TL	AGG GAM-Kalman TL	AGG Kalman TL
Transfer of GAM	No	No	Yes	Yes	No
Cost	$C_1 \times m_T$	$C_1 \times m_T$	$C_1 \times n_1<< C_1 \times m_T$	$C_1 \times n_2<< C_1 \times m_T$	$C_1 \times m_T$
Transfer of Kalman variances	–	No	–	Yes	Yes
Cost	–	$C_2 \times m_T$	–	$C_2 \times n_2<< C_2 \times m_T$	$C_2 \times n_3<< C_2 \times m_T$
Model type	${\mathscr {M}}_{k,0,k}$	${\mathscr {M}}_{k,k,k}$	${\mathscr {M}}_{i,\emptyset ,k}$	${\mathscr {M}}_{i,i,k}$	${\mathscr {M}}_{k,j,k}$
Total cost	$C_1 \times m_T$	$(C_1 + C_2) \times m_T$	$C_1 \times n_1$	$(C_1 + C_2) \times n_2$	$C_1 \times m_T + C_2 \times n_3$

## Data and model presentation

Firstly, French local electricity consumption and explanatory variables are introduced. The models are then detailed in the practical point of view: definition of GAMs formula, training of Kalman filter and construction of the aggregation methods. To guarantee data confidentiality, substations’ identities are not provided, and the electricity loads represented in the different figures are normalized by the average load.

### Presentation of the data

#### Electricity load data

**Figure 2:**
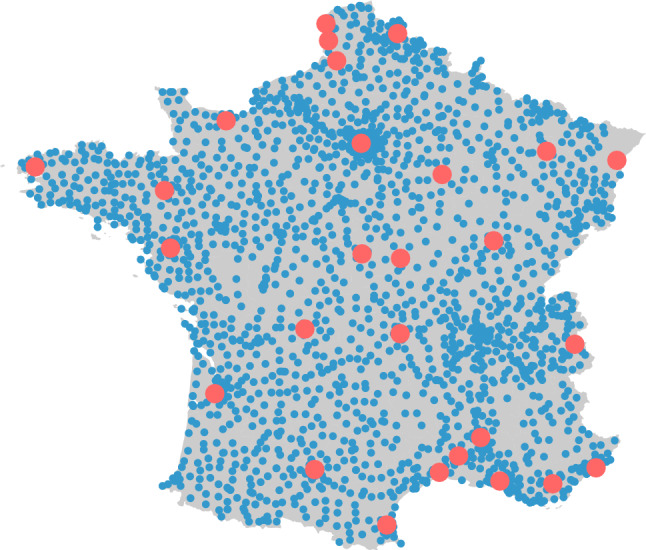
Location of the 1344 substations (in blue) and 27 weather stations (in red). The map has been created thanks to the R packages ggplot2 (version 3.4.2, https://cran.r-project.org/web/packages/ggplot2/index.html) and maps (version 3.4.1, https://cran.r-project.org/web/packages/maps/index.html).

The data are provided by Enedis, the operator in charge of the electric power distribution in France. The data are composed of 1344 time series, each of which is the electricity consumption of one substation represented in blue in Fig. 2.

**Figure 3:**
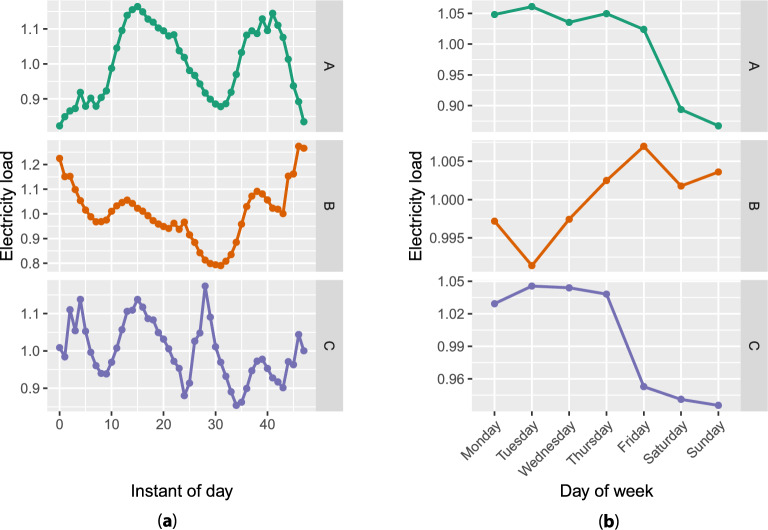
Electricity loads in March for three substations: (**a**) averaged daily load and (**b**) averaged weekly load. Substation A has the expected daily and weekly patterns: the load is ordinarily high during daylight, especially when people are at home, low during the night, and similar during weekdays, with a drop during weekends. On the other hand, substation B has opposed daily behavior at night, and load is increasing during the week and maximal during weekends. Substation C shows a high load in the mid-afternoon, and load is still important at night. Concerning its weekly load, it drops Friday in addition to the weekend.

They cover metropolitan France and reflect thus its local electricity consumption. Forecasting each substation’s consumption involves $m_T = 1344$ forecasting tasks. The data are available from June 1st, 2014, to December 31st, 2021, with a 30-minute temporal resolution. Although most of the 1344 time series present classical temporal and meteorological patterns, there are some counter-intuitive and contrary variations. Figure 3 is a comparison between one substation with basic behavior and two substations with unusual behaviors.

An operational constraint on data availability is fixed: the load of the previous day, labeled as $D-1$, becomes available on day *D* for forecasting day $D+1$. Given the temporal resolution of the data, 48 forecasts are made for the 48 instants of day. The forecast for an instant is made once the load of the instant of the previous day is available, independently of the instant. In other words, the forecasting strategy is sequential or online. The choice of forecasting context is crucial as it impacts the quality of the forecasts.

#### Explanatory variables

**Figure 4:**
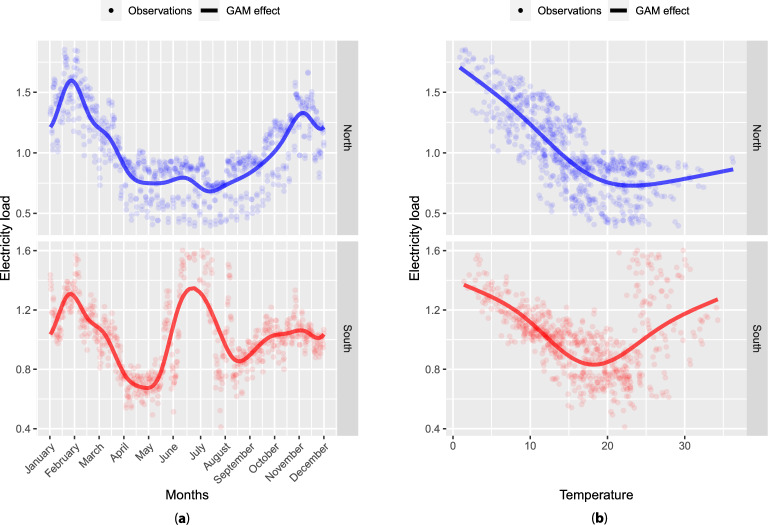
Dependence of 2015 electricity consumption at 6 p. m. to (**a**) time of year and (**b**) temperature. Blue values for a substation in the north of France and red values for one in the south of France.

For explanatory variables, meteorological and calendar variables are chosen, a common practice in electricity load forecasting. Weather variables are obtained from Météo-France, the French public institution of meteorology and climatology. They are composed of temperature, cloudiness, and wind measurements from 27 weather stations. Temperature is represented in degrees Celsius, cloudiness is the amount of cloud cover measured in octas, and wind is measured 10 meters above the ground and is expressed in meters per second. The weather stations are unequally spread over metropolitan France and are represented in red in Fig. 2. Weather data are available in the same range as electricity consumption with a 3 hours temporal resolution. These data are transformed into 30 minutes of temporal resolution thanks to linear interpolation. Temperature is highly correlated to electricity load with a different impact for cold and hot regions, as shown in Fig. 4. The two patterns are due to the use of electrical heating and air-conditioning systems in France, which are highly electric consuming. To generate localized datasets, each substation and its nearest weather station are paired, creating substation-specific datasets with local weather data.

An aspect to consider within the forecasting context is the availability of weather variables during the prediction process. In operational conditions, the weather variables used in the models are forecasted since future weather is not yet available. On the other hand, this article operates under the assumption that, on day *D*, the weather variables for day $D+1$ are already available. While this setup is clearly unfeasible operationally, it holds significance for two main reasons. First, in the context of day-ahead forecasting, the weather inputs would be weather forecasts of the next day, which can be assumed to be accurate and thus close to the actual weather of the next day. Second, the objective is to disentangle the forecasting error induced by the quality of the weather forecast, an external factor beyond our control, from the error linked to the forecasting model itself. In a similar setting, weather data availability has been discussed with a highlight on the importance of a good temperature forecast in operational conditions^13^.

Concerning calendar variables, they gather indicators of school holidays by region, bank holidays, and working days, as well as the instant of the day, time of year, and days of the week. French regions are divided into three zones, and each zone has its own calendar for school holidays. This factor is taken into consideration when constructing our models.

#### Time segmentation

**Figure 5:**
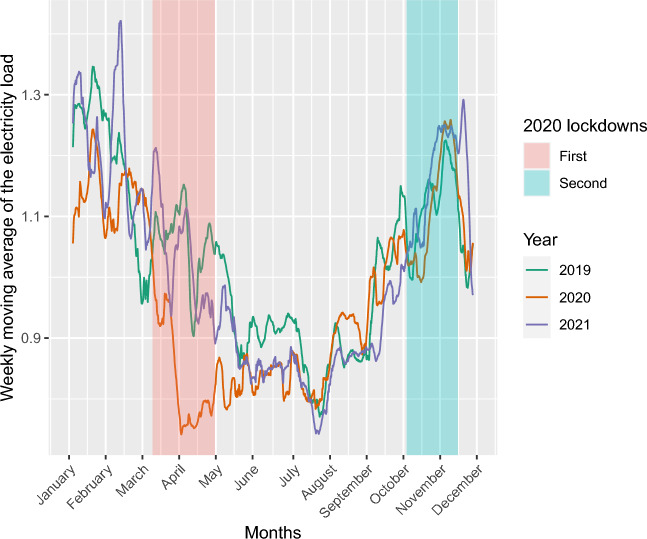
Weekly moving average of the electricity load of one substation in 2019, 2020, and 2021.

The data ranges from June 2014 to December 2021. Training and validation of the models are performed on the data up to December 31st, 2019, and test is done afterwards. The test set includes COVID-19 observations and three lockdown periods in France. The second and third lockdowns are considered as normal periods because the electricity consumption strongly varies only during the first lockdown, see Fig. 5. Three validation periods are thus chosen: 2020 out of the first lockdown, the first lockdown (from March 16th, 2020, to May 11th, 2020), and 2021.

### Generalized additive model formula

To build a GAM, one must determine its formula: which explanatory variables and spline bases to use. To do so, 2018 is used as a test period, and the different tests have been achieved with a forward-backward heuristic. An assumption is made that a single GAM formula could be used for all target forecasting tasks. Indeed, although substations’ behaviors are different, they can be explained by the same explanatory variables. Finally, a GAM is applied by instant of the day; that is, 48 GAMs are optimized for each forecasting task. The unique following GAM formula is obtained:

7 $$\begin{aligned} \begin{aligned} y_t =&\sum _{i=1}^5 \alpha _i 1_{DayType_t = i} + \sum _{i=1}^2 \beta _i 1_{BankHoliday_t = i} + \sum _{i=1}^2 f_i(ToY_t) 1_{WorkingDay=i} + f_3(Trend_t) + f_4(Temp_t) + f_5(Temp95_t) \\&+ f_6(Temp99_t) + f_7(TempMin_t, TempMax_t) + \gamma _1 Load2D_t + \gamma _2 Load1W_t + \varepsilon _t, \end{aligned} \end{aligned}$$

where at each time step *t*,

- $y_t$ is the electricity load for the considered instant,
- $DayType_t$ is a categorical variable indicating the type of the day. There are five categories: Monday, Tuesday to Thursday, Friday, Saturday, and Sunday,
- $BankHoliday_t$ is a binary variable indicating whether the day *t* is a bank holiday or a school holiday depending on the region of the relevant substation,
- $ToY_t$ is the time of year whose values grow linearly from 0 on the 1st of January midnight to 1 on the 31st of December 23h30,
- $WorkingDay_t$ is a binary variable indicating whether the day *t* is a working day, i.e., not a weekend day or a bank holiday,
- $Trend_t$ is the number of the current observation,
- $Temp_t$ is the measured temperature of the closest weather station,
- $Temp95_t$ and $Temp99_t$ are exponentially smoothed $Temp_t$ variable of factor $\alpha = 0.95$ and 0.99. E.g. for $\alpha = 0.95$ at a given time step *t*, $Temp95_t = \alpha Temp95_{t-1} + (1 - \alpha ) Temp_t$,
- $TempMin_t$ and $TempMax_t$ are the minimal and maximal value of $Temp_t$ at the current day,
- $Load2D_t$ and $Load1W_t$ are loads of 2 days before and the load of the week before,
- $\varepsilon _t$ is Gaussian noise with 0 mean and constant variance.

This model is a short-term model in terms of electricity past consumption availability. It is thus called ST GAM. A model called MT GAM is also considered, which is the same model without the $Load2D_t$ and $Load1W_t$ effects.

The case where $\varepsilon _t$ is an auto-correlated error term is also examined. In that case, an ARIMA (autoregressive integrated moving average) model is chosen by selecting the best model with AIC criterion in the family of ARIMA(p,d,q). Correcting residuals of mid and short-term GAM with an ARIMA model achieves the same performance; therefore, the more straightforward mid-term formula is chosen. It can be explained by the redundancy of correcting auto-correlated residuals with an ARIMA model and the linear lag terms in the short-term GAM. The following model is called MT GAM + ARIMA.

Thin plate spline basis with low dimensions represent all the effects except $f_1$ and $f_2$. Indeed, time of year has a cyclic impact (see Fig. 4 (**a**)); therefore, a cyclic cubic splines basis of dimension 20 is employed. GAM effects estimation is done using the Generalized Cross Validation criterion and takes a few tens of seconds in practice. Thus, it is computationally reasonable to train individual GAM for many forecasting tasks. Finally, the GAMs are trained using R and the package mgcv^39^.

### Kalman filtering

This section examines Kalman filtering adaptation of the previous short-term GAM. In the static setting, one can apply one individual adapted GAM to each forecasting task as no Kalman variances estimation is necessary. It is called GAM + Kalman Static. It is a traditional machine learning context where the model is optimized on the same data set to be forecasted.

Execution time is essential in the dynamic setting. It is an unavoidable operational constraint, and the decision is made to calculate Kalman variances on a subsample of the 1344 substations. This subsample is chosen to represent the variety of the difficulty of forecasting. First, the 1344 substations are sorted according to the performance (NMAE, see section “Experiments”) of their short-term GAM predictions for 2018. The 44 substations corresponding to the 44 worst performances are put aside as they won’t provide interesting experts. This allows dividing the 1300 substations into 13 groups of 100 substations each. A sample of 6 substations is then randomly drawn from each group of 100. Ultimately, a subsample of 78 representative substations is obtained, reflecting the forecasting difficulty. The parallel computing of the 78 corresponding sets of Kalman variances on a 36-core virtual machine on 2014–2018 data takes about 1.6 days. The computation of 1344 individual Kalman variances would thus last about 28 days in a similar setting. The parameters of this sampling method have not been optimized for two reasons. First, it would require a significant amount of computational time and associated emissions, which contradicts the frugal philosophy of the article. Second, it is assumed that the sampling method has a very low impact on the final quality of the forecast compared to other model parameters, like the number of experts used in the aggregations.

Two sets of Kalman variances are calculated on the same substations subsample: one on 2014–2018 data and the other on 2014–2019 data. The first set is used to set the aggregation hyper-parameters $n_i$ with 2019 as the validation set, and the second set is used to forecast 2020–2021 as the test set. Thus, there are at most 78 GAMs adapted by their individual Kalman variances. In that case, the corresponding model is called GAM + Kalman Dynamic, and it is shown in the Experiments section that the aggregation methods achieve equivalent performances.

Kalman filters estimation and application are achieved using R and the package viking^40^.

### Aggregation

A reminder on the three aggregation methods can be found in the Aggregation models section and Table 1. This section focuses on their application.

**Figure 6:**
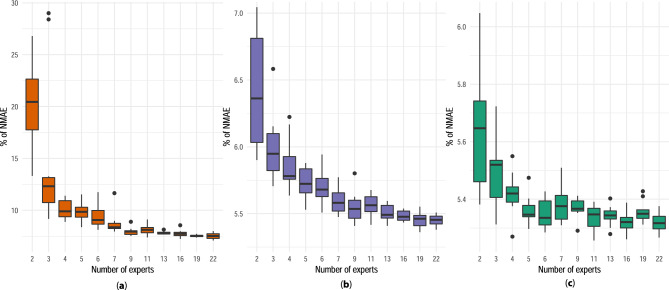
Grid search of the number of experts: (**a**) $n_1$ for AGG GAM TL, (**b**) $n_2$ for AGG GAM-Kalman TL, and (**c**) $n_3$ for AGG Kalman TL.

For each aggregation method, the unique hyper-parameter is $n_i$, the number of experts in the aggregation, which is also the number of sources in the transfer learning task. As said previously, $m_S$ should be the smallest possible compared to $m_T = 1344$. A grid search is performed on 2019 data as the validation set. 10 forecasts of 182 representative substations are carried out for different values of $n_i$ and the 10 corresponding medians are then computed. The 182 representative substations are obtained in the same way as the 78 substations used to optimize Kalman variances. The results are presented in Fig. 6. The aggregation becomes gradually robust with the increase in the number of experts under the randomness of substations selection to compute GAM effects and Kalman variances. Moreover, there is an elbow phenomenon after $n_1 = n_2 = 9$ for AGG GAM TL and AGG GAM-Kalman TL, and after $n_3 = 6$ for AGG Kalman TL. These values of $n_i$ are very little compared to $m_T = 1344$.

## Experiments

In this section, a model analysis is provided thanks to visualization plots and performances on substations data sets are compared using a comparative metric.

### Model dynamics

Aggregation methods are first analyzed at the experts’ scale and then at the GAM effects’ scale.

**Figure 7:**
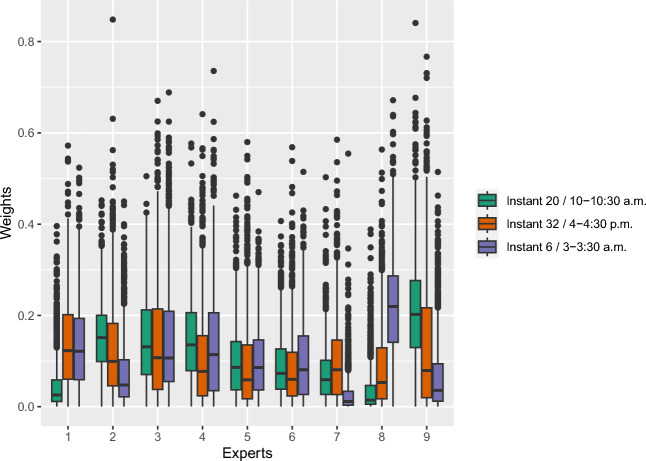
Boxplot representation of the weights of the 1344 AGG GAM-Kalman TL aggregations for three instants of the day. 2021 target forecasting tasks.

At the experts’ scale, one might want to know which expert is most important in the mixture and when. To do so, the distribution of the weights of each expert is studied over all the forecasts produced for each substation. Indeed, the higher the weight, the more important the corresponding expert is in the forecast. One expert can be important in the forecast for specific observations and not for others. To give an example, Fig. 7 represents boxplots of the weights of the 1344 AGG GAM-Kalman TL aggregations for three interesting instants of the day where the consumption behaviors are very different. From this representation, it can be deduced that experts 3, 4, 5, and 6 are as essential during the three instants of the day, with medians near the uniform weight of 1/9. On the other hand, the other experts’ weights vary more according to the instant of the day: for each instant, each expert is of low importance at least once (weight close to 0) and of great importance at least once (weight close to 0.5 or bigger).

**Figure 8:**
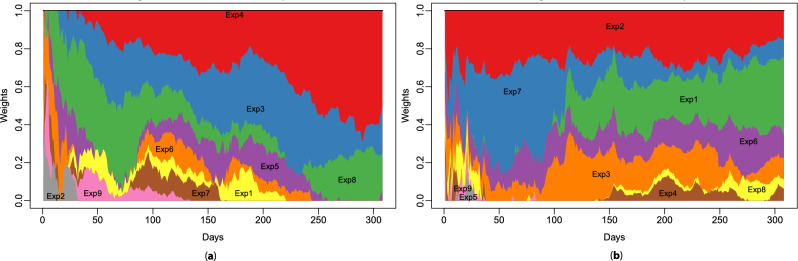
Weights associated with the experts of AGG GAM-Kalman TL aggregation at midday for (**a**) substation D and (**b**) substation E. Experts are denoted Exp*i*, and the forecasting period is 2020 out of the first lockdown.

Another way of analysing the importance of experts is by focusing on each forecast to see which experts are used and when in an aggregation. Figure 8 displays the evolution of weights from AGG GAM-Kalman TL aggregation for two specific substations at midday. The same previous conclusions are observed: some experts have very little impact on the aggregation, while others are important. The new information these figures provide is the evolution of experts’ importance during aggregation. For example, in Fig. 8a, Exp4’s weight increases and becomes of paramount importance, with the weight being superior to 1/2 for the last observations. In Fig. 8b, Exp7 vanishes rapidly while the impact of Exp1 increases. Exp5 and Exp9 contribute very little to the first observations and are quickly absent in the aggregation.

**Figure 9:**
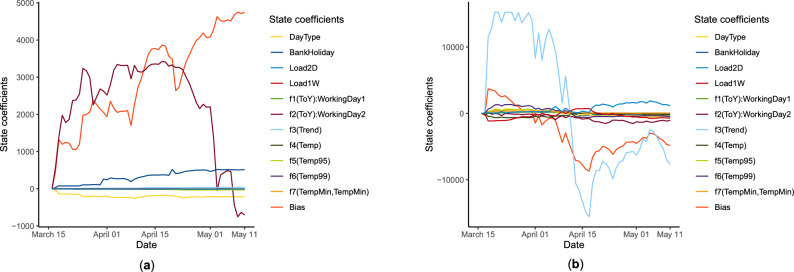
Evolution of (**a**) state coefficients of a Kalman TL model $(\hat{\theta }_{t,d} - \hat{\theta }_{0,d})_{d=1}^D$ and (**b**) hybrid state coefficients of a AGG Kalman TL aggregation $(\widetilde{\theta }_{t,d} - \widetilde{\theta }_{0,d})_{d=1}^D$, during the first lockdown and for the same target forecasting task.

At the GAM effects’ scale, a visualization of the evolution of state coefficients from Kalman filtering is done; precisely, by plotting the *D* curves corresponding to the *D* GAM effects. Figure 9 (**a**) provides an example with the evolution of state coefficients of an adapted GAM ${\mathscr {M}}_{k,k,k}$ during the first lockdown. It can be observed that the coefficients associated with Bias and $f_2(ToY)$, which is the effect modeling the time of year during working days, are more significant compared to others and evolve rapidly. Concerning the bias coefficients, they evolve to balance the gap between the source load used to train the GAM and the target load, which can be of different scales. Finally, some state coefficients evolve significantly, like *BankHoliday* or *DayType*, while others seem time-invariant.

To extend this visual diagnosis tool to the case of AGG Kalman TL, one can express the aggregation as a GAM adapted by hybrid state coefficients $(\widetilde{\theta }_{t,d})_{d=1}^D$:

8 $$\begin{aligned} \hat{y}_t = \sum _{k=1}^E \hat{p}_{e,t} \hat{y}_{e,t} = \sum _{e=1}^E \hat{p}_{e,t} \sum _{d=1}^D \hat{\theta }_{t,d,e} f_d (x_{t,d}) = \sum _{d=1}^D \left( \sum _{k=1}^E \hat{p}_{e,t} \hat{\theta }_{t,d,e} \right) f_d(x_{t,d}) = \sum _{d=1}^D \widetilde{\theta }_{t,d} f_d(x_{t,d}), \end{aligned}$$

where at time *t*, $\widetilde{\theta }_{t,d} = \sum _{e=1}^E \hat{p}_{e,t} \hat{\theta }_{t,d,e}$ denotes the hybrid state coefficients, *E* the number of experts, *D* the number of GAM effects, $\hat{p}_{e,t}$ the weight associated with expert *e*, and $\hat{\theta }_{t,d,e}$ the adaptation coefficient associated with the GAM effect $f_d$ of expert *e*. This new expression allows for gathering information on both adaptation and aggregation and improving the interpretation of the whole model. Figure 9 (**b**) represents the evolution of these hybrid state coefficients during the first lockdown for the same target forecasting task as in Fig. 9a. One can therefore compare the Kalman coefficients $(\hat{\theta }_{t,d,e})_{d=1}^D$ of an individual model ${\mathscr {M}}_{k,k,k}$ and the hybrid state coefficients $(\widetilde{\theta }_{t,d})_{d=1}^D$ calculated from $n_3 ~ {\mathscr {M}}_{k,j,k}$ models coefficients. Both types of coefficients differ in amplitude and dynamic. Bias coefficients are still important compared to the others and evolve rapidly, but it’s not the case for $f_2$ coefficients.

When considering any aggregation method, the entire mixture can be expressed as a GAM using a linear combination of GAM effects. The new coefficients of the spline basis are composed of the basic coefficients of the spline basis, the adaptation coefficients, and the aggregation weights.

### Numerical results

To compute numerical performances of the models, the normalized mean absolute error (NMAE) is used. It is defined for a time series $(y_t)_t$, a forecast $({\hat{y}}_t)_t$ and a test set ${\mathscr {T}}$ by:

9 $$\begin{aligned} \text {NMAE} =\frac{\sum _{t\in {\mathscr {T}}} |y_t - \hat{y}_t|}{\sum _{t\in {\mathscr {T}}} |y_t|}. \end{aligned}$$

It is the mean absolute error between the ground truth and the forecast, normalized by the absolute mean of the time series. This metric allows to compare forecasts of different scales, which is necessary in the case study because the individual NMAE are computed for each time series. The NMAE is chosen rather than the mean absolute percentage error because the time series are sometimes very close to 0. More precisely, the percentage of NMAE is examined by multiplying it by 100.

**Table 2:** Numerical performances of the 1344 forecasting tasks in normalized mean absolute error (NMAE) (%). Q1 means the first quartile, Q2 the median, and Q3 the third quartile.

Method	2020 out of the first lockdown			First lockdown			2021		
Quartiles	Q1	Q2	Q3	Q1	Q2	Q3	Q1	Q2	Q3
ST GAM	5.35%	6.50%	8.69%	7.31%	9.37%	13.30%	6.29%	7.87%	11.00%
MT GAM	6.67%	8.34%	11.50%	9.44%	12.50%	18.70%	8.17%	10.70%	16.30%
MT GAM + ARIMA	4.74%	5.73%	7.53%	6.28%	7.91%	10.70%	5.13%	6.17%	8.37%
GAM + Kalman Static	5.00%	5.98%	7.90%	6.96%	8.84%	12.20%	5.26%	6.25%	8.32%
AGG GAM TL	6.21%	7.67%	10.90%	8.02%	10.10%	14.40%	6.23%	7.58%	10.80%
AGG GAM-Kalman TL	4.29%	5.11%	6.68%	5.26%	6.37%	8.20%	4.44%	5.33%	6.98%
AGG Kalman TL	4.25%	5.08%	6.70%	5.43%	6.53%	8.56%	4.39%	5.26%	6.87%
Expert Oracle	4.40%	5.24%	6.78%	5.01%	5.98%	7.67%	4.58%	5.47%	7.07%
Convex Oracle	4.11%	4.90%	6.32%	4.52%	5.41%	6.97%	4.26%	5.12%	6.67%

The performances of the individual and aggregation models displayed in Table 2 are first compared. The improvements in performance are referenced with respect to the three respective validation periods.

It can be noted that the information on the past electricity load added in the GAM formula is of great importance, with a significant improvement between ST GAM and MT GAM (1.84%, 2.83%, and 3.13% for the medians). There is also a gain when MT GAM residuals are modeled with an ARIMA model: there is an improvement between ST GAM and MT GAM + ARIMA (0.77%, 1.46%, and 1.70% for the medians). GAM + Kalman Static provides slightly weaker performances than MT GAM + ARIMA. The latter is thus the individual benchmark to beat.

Concerning the aggregation methods, AGG GAM TL shows the worst performances. However, this model is still interesting as its performances are close to the ST GAM performances while its computational cost is much lower. On the other hand, the two other aggregation methods show excellent and close performances. AGG Kalman TL is better than AGG GAM-Kalman TL outside of the first lockdown (improvement in the median of 0.03% and 0.07%), while it is the opposite for the first lockdown (decrease of 0.16%). The gap between these two last models is not significant. As AGG GAM-Kalman TL is less complex than AGG Kalman TL, it is considered as the best compromise between forecasting performance and computational cost. Compared to MT GAM + ARIMA, the best individual model, its median performances improve by 0.62%, 1.54%, and 0.84%.

**Figure 10:**
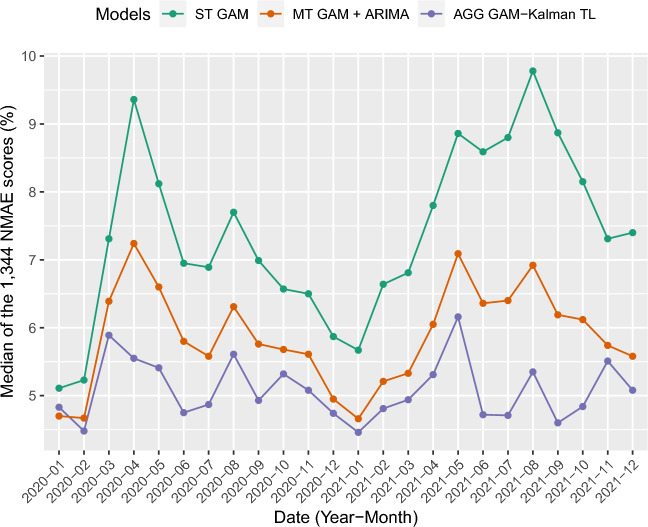
Median of the 1344 percentages of NMAE by months in 2020 and 2021 of three models : ST GAM, MT GAM + ARIMA and AGG GAM-Kalman TL.

Figure 10 shows the evolution of the median errors over months. Three models are considered : the initial model, the best individual model and the best aggregation. It indicates that, except for the month of January 2020, the final model is consistently better than the benchmark.

As explained in the “Transfer learning using aggregation of experts” section, aggregation algorithms aim at beating two oracles: the best-fixed expert and the best-fixed convex combination of experts. They are called Expert Oracle and Convex Oracle, respectively, and their performances are computed for AGG GAM-Kalman TL. As displayed by Table 2, the aggregation competes with the best-fixed expert and approaches the best-fixed combination of experts. The same can be said for the other two aggregation methods.

Concerning the test periods, one can see that the 2020 forecasts worsen during the first French lockdown with a downgrade of 2.18% between the two MT GAM + ARIMA median performances. AGG GAM-Kalman TL and AGG Kalman TL present lower downgrades: 1.26% and 1.45% in the median, respectively; that is, the online nature of Kalman filtering and aggregation of experts allow adaptation to the extreme change in electricity consumption. On the other hand, ST GAM and MT GAM performances worsen in 2021 as these models are not updated while the consumption behavior evolves. The downgrades are significant: 1.37% and 2.36% in the median, respectively. The performance gaps between 2020 out of the first lockdown and 2021 are tighter for adaptative models, especially for the two best aggregation methods: 0.22% and 0.18% in the median, respectively. Once again, this shows that the aggregation methods adapt well to the data distribution evolution.

**Table 3:** Numerical performances of 69 representative forecasting tasks in normalized mean absolute error (NMAE) (%). Q1 means the first quartile, Q2 the median, and Q3 the third quartile.

Method	2020 out of the first lockdown			First lockdown			2021		
Quartiles	Q1	Q2	Q3	Q1	Q2	Q3	Q1	Q2	Q3
GAM + Kalman Dynamic	4.45%	5.36%	7.02%	5.75%	7.50%	10.10%	4.55%	5.22%	7.79%
AGG GAM TL	6.25%	7.79%	11.30%	8.65%	11.10%	14.90%	6.05%	7.63%	13.10%
AGG GAM-Kalman TL	4.23%	5.36%	7.11%	5.64%	6.91%	8.86%	4.27%	5.41%	7.73%
AGG Kalman TL	4.39%	5.48%	7.15%	6.03%	7.67%	10.10%	4.48%	5.42%	7.89%

**Figure 11:**
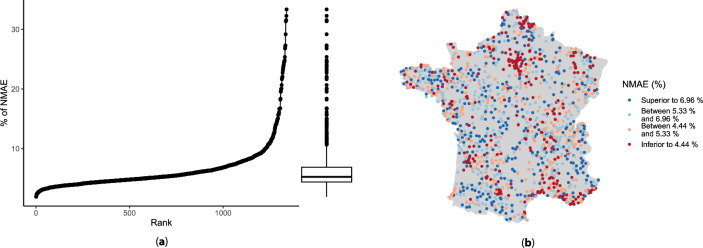
Analysis of AGG GAM-Kalman performance of the 1344 forecasting tasks during 2021: (**a**) shows the sorted percentages of NMAE and (**b**) represents their spatial distribution. The dots correspond to substations locations and the four categories of the legend correspond to the first quartile (4.44%), median (5.33%), and the third quartile (6.96%). The map has been created thanks to the R packages ggplot2 (version 3.4.2, https://cran.r-project.org/web/packages/ggplot2/index.html) and maps (version 3.4.1, https://cran.r-project.org/web/packages/maps/index.html).

Table 3 provides performances of 69 forecasting tasks. Precisely, they correspond to the 78 substations used to optimize the set of Kalman variances reduced of the 9 substations providing the experts involved in the three aggregations. This allows the comparison of the aggregation methods with GAM + Kalman Dynamic. As previously said, GAM + Kalman Dynamic achieves great performances forecasting the national electricity load and is thus a competitive model and the best one among the individual models. However, it requires large amounts of time to estimate. It is remarkable that AGG GAM-Kalman TL and AGG Kalman TL perform similarly while being much less computationally costly.

Finally, the distribution of the 1344 NMAE scores is depicted in Fig. 11a and the geographical distribution in Fig. 11b. It is interesting to notice that the best forecasted substations are close to urban areas like Paris, Marseille, Lille, or Bordeaux, whereas the worst forecasted are located in rural areas.

## Conclusion

In this paper, a frugal method is proposed to forecast multiple electricity loads. The estimation of an individual model per time series is avoided, resulting in a scalable methodology based on the aggregation of experts with limited computational resources. The chosen experts are GAMs adapted by Kalman filtering, which have performed very well for electricity consumption forecasting. The aggregation methods allow the transfer of GAMs and Kalman filters separately or simultaneously. This paper demonstrates that they provide good forecasts compared to competitive models, especially during the first French lockdown. Moreover, they don’t need human intervention nor expertise and are thus simple to use. The method is frugal in terms of parameter estimation, and the computational cost has been discussed extensively. Finally, it benefits from the interpretability of GAM and the aggregation of experts.

There are various ways to extend the work presented in this paper. First, the random selection of the time series used to train GAMs and Kalman variances can be improved. Clusters of substations may be identified according to some characteristics (geographic, weather, type of consumers) and one representative selected per cluster. A second way of improvement is the inclusion of new explanatory variables at the same local scale : geo-tracking and communication data reflect human behavior and are therefore helpful for electric consumption forecasting^33^.

This paper focused on learning models on single time series, then transferring them to other ones; it may be possible to transfer models trained on several substations. One could easily train a GAM jointly on several time series (concatenating data sets), however, it is not trivial to learn the Kalman hyper-parameters using several substations due to the sequential structure of state-space models.

Finally, the computational complexity of the method introduced essentially depends on the estimation of a fixed number of models; our objective in doing so is to obtain a very scalable forecasting method. Therefore one could apply the method to a larger data set, for instance the electrical consumption at a finer granularity.

## Data Availability

The data that support the findings of this study are available from Enedis but restrictions apply to the availability of these data, which were used under license for the current study, and so are not publicly available. Data are however available from the authors upon reasonable request and with permission of Enedis.

## References

[1] Hong T, Pinson P, Fan S (2014). Global energy forecasting competition 2012. Int. J. Forecast..

[2] Hong T (2016). Probabilistic energy forecasting: Global energy forecasting competition 2014 and beyond. Int. J. Forecast..

[3] Hong T, Xie J, Black J (2019). Global energy forecasting competition 2017: Hierarchical probabilistic load forecasting. Int. J. Forecast..

[4] Huang S-J, Shih K-R (2003). Short-term load forecasting via arma model identification including non-gaussian process considerations. IEEE Trans. Power Syst..

[5] Chodakowska E, Nazarko J, Nazarko Ł (2021). Arima models in electrical load forecasting and their robustness to noise. Energies.

[6] Jalil N, Ahmad M, Mohamed N (2013). Electricity load demand forecasting using exponential smoothing methods. World Appl. Sci. J..

[7] Aguilar Madrid E, Antonio N (2021). Short-term electricity load forecasting with machine learning. Information.

[8] Lloyd JR (2014). Gefcom 2012 hierarchical load forecasting: Gradient boosting machines and gaussian processes. Int. J. Forecast..

[9] Park DC, El-Sharkawi M, Marks R, Atlas L, Damborg M (1991). Electric load forecasting using an artificial neural network. IEEE Trans. Power Syst..

[10] Ryu S, Noh J, Kim H (2016). Deep neural network based demand side short term load forecasting. Energies.

[11] Wood SN (2017). Generalized Additive Models: An Introduction with R.

[12] Pierrot, A. & Goude, Y. Short-term electricity load forecasting with generalized additive models. *Proceedings of ISAP power* **2011** (2011).

[13] Goude Y, Nedellec R, Kong N (2014). Local short and middle term electricity load forecasting with semi-parametric additive models. IEEE Trans. Smart Grid.

[14] Fasiolo M, Wood SN, Zaffran M, Nedellec R, Goude Y (2021). Fast calibrated additive quantile regression. J. Am. Stat. Assoc..

[15] Fan, S. & Hyndman, R. J. Forecasting electricity demand in australian national electricity market. In *2012 IEEE Power and Energy Society General Meeting*, 1–4 (IEEE, 2012).

[16] de Vilmarest, J. *Modèles espace-état pour la prévision de séries temporelles. Application aux marchés électriques.* Ph.D. thesis, Sorbonne Université (2022).

[17] Obst D, de Vilmarest J, Goude Y (2021). Adaptive methods for short-term electricity load forecasting during Covid-19 lockdown in France. IEEE Trans. Power Syst..

[18] de Vilmarest J, Goude Y (2022). State-space models for online post-covid electricity load forecasting competition. IEEE Open Access J. Power Energy.

[19] Cesa-Bianchi N, Lugosi G (2006). Prediction, Learning, and Games.

[20] Gaillard P, Goude Y, Antoniadis A, Poggi J-M, Brossat X (2015). Forecasting electricity consumption by aggregating experts; how to design a good set of experts. Modeling and Stochastic Learning for Forecasting in High Dimensions.

[21] Miller C (2020). The ashrae great energy predictor iii competition: Overview and results. Sci. Technol. Built Environ..

[22] Makridakis S, Spiliotis E, Assimakopoulos V (2018). The m4 competition: Results, findings, conclusion and way forward. Int. J. Forecast..

[23] Makridakis S, Spiliotis E, Assimakopoulos V (2022). Predicting/hypothesizing the findings of the m5 competition. Int. J. Forecast..

[24] Januschowski T (2020). Criteria for classifying forecasting methods. Int. J. Forecast..

[25] Buonanno A (2022). Global vs. local models for short-term electricity demand prediction in a residential/lodging scenario. Energies.

[26] Montero-Manso, P. & Hyndman, R. J. Principles and algorithms for forecasting groups of time series: Locality and globality. *Int. J. Forecast.* **37**, 1632–1653. arXiv:2008.00444 (2021).

[27] Bottou, L. & Bousquet, O. The tradeoffs of large scale learning. *Advances in neural information processing systems* **20** (2007).

[28] Hazan, E. *et al.* Introduction to online convex optimization. *Found. Trends Optim.* **2**, 157–325, arXiv:1909.05207 (2016).

[29] García-Martín E, Rodrigues CF, Riley G, Grahn H (2019). Estimation of energy consumption in machine learning. J. Parallel Distrib. Comput..

[30] Carvalho DV, Pereira EM, Cardoso JS (2019). Machine learning interpretability: A survey on methods and metrics. Electronics.

[31] Kaissis GA, Makowski MR, Rückert D, Braren RF (2020). Secure, privacy-preserving and federated machine learning in medical imaging. Nat. Mach. Intell..

[32] Evchenko, M., Vanschoren, J., Hoos, H. H., Schoenauer, M. & Sebag, M. Frugal machine learning. arXiv preprint arXiv:2111.03731 (2021).

[33] Gaucher, S., Goude, Y. & Antoniadis, A. Hierarchical transfer learning with applications for electricity load forecasting. arXiv preprint arXiv:2111.08512 (2021).

[34] Zhuang F (2020). A comprehensive survey on transfer learning. Proceedings of the IEEE.

[35] Gaillard, P., Stoltz, G. & van Erven, T. A second-order bound with excess losses. In *Proceedings of The 27th Conference on Learning Theory*, vol. 35 of *Proceedings of Machine Learning Research* (eds. Balcan, M. F., Feldman, V. & Szepesvári, C.) 176–196 (PMLR, Barcelona, 2014).

[36] Gaillard P, Goude Y, Nedellec R (2016). Additive models and robust aggregation for gefcom2014 probabilistic electric load and electricity price forecasting. Int. J. Forecast..

[37] Gaillard, P. & Goude, Y. opera: Online prediction by expert aggregation. https://CRAN.R-project.org/package=opera, R package version (2016).

[38] Kalman RE (1960). A new approach to linear filtering and prediction problems. J. Basic Eng..

[39] Wood, S. Package ‘mgcv’. https://cran.r-project.org/package=mgcv, R package version **1**, 729 (2015).

[40] de Vilmarest, J. Viking: State-space models inference by Kalman or Viking. https://cran.r-project.org/package=viking, R package version (2022).

